# Case of Colica Pictonum, Produced by the White Lead Treatment of a Severe Scald

**Published:** 1857-07

**Authors:** G. A. Kunkler

**Affiliations:** Madison, Ia.


ART. VI.—*Case of Colica Pictonum produced by the White Lead Treatment of a severe Scald.* By G. A. KUNKLER, M.D., of Madison, Ia

THE following case may not prove without interest, in showing how readily the toxic effects of lead may manifest themselves in certain persons, after its external application to a denuded surface.

Kate B., an Irish servant girl, scalded her arm by a kettle of boiling water falling upon it during the latter part of last December. The injury extended from the elbow over the whole forearm and hand, and was followed by extensive vesication. After the contents of the vesicles were evacuated, by puncturing them with a needle, the common white paint, of the consistency of cream, was applied freely with a camel's hair brush, the parts covered with cotton, and a roller lightly applied over the whole. The accident occurred early in the morning; and on the following day, the dressings having become dry, were renewed. On the evening of the third day, I saw the patient again, and then found her laboring under unmistakable symptoms of *colica saturnina*, such as acute abdominal pain, retraction of the umbilicus, constipation, and *slight discoloration of the gums.* The burn was doing well; under the use of opium, purgatives, &c., she was rapidly relieved. The use of the paint was discontinued, and the linseed oil and lime water mixture substituted, from the use of which the burn got well. During the past five years, I have used the white paint in a number of cases of burns and scalds of every imaginable description, without ever noticing any injurious result; my attention having been first called to it by a paper of Professor Gross, in vol. iii of the Transactions of the Am. Med. Assoc. In one case, in particular, where both the lower extremities of a child, five years of age, were badly scalded from the hips down to the feet, the white paint was applied several times over the whole surface, without any injurious result whatever; the convalescence being extraordinarily rapid.

In the first-mentioned case, I was particular to investigate whether lead in any shape could have entered the system through any other channel; but was soon satisfied that this was altogether improbable, and I am, therefore, firmly convinced that the colic was due entirely to the absorption of the lead from the burnt surface. [This is the only well-authenticated case of colicky symptoms resulting from the local use of white lead in burns and scalds that we remember to have ever heard of, notwithstanding its very general employment. The occurrence of such a case is therefore to be regarded only as a great rarity, and should not be urged as an objection to the remedy. When effects of this sort occur, they may be promptly relieved by the internal administration of aromatic sulphuric acid in repeated doses.—ED.]

